# Unifying turbulent dynamics framework distinguishes different brain states

**DOI:** 10.1038/s42003-022-03576-6

**Published:** 2022-06-29

**Authors:** Anira Escrichs, Yonatan Sanz Perl, Carme Uribe, Estela Camara, Basak Türker, Nadya Pyatigorskaya, Ane López-González, Carla Pallavicini, Rajanikant Panda, Jitka Annen, Olivia Gosseries, Steven Laureys, Lionel Naccache, Jacobo D. Sitt, Helmut Laufs, Enzo Tagliazucchi, Morten L. Kringelbach, Gustavo Deco

**Affiliations:** 1 Computational Neuroscience Group, Center for Brain and Cognition, Department of Information and Communication Technologies, Universitat Pompeu Fabra, Barcelona, Catalonia Spain; 2 Universidad de San Andrés, Buenos Aires, Argentina; 3 Institut du Cerveau et de la Moelle épinière, ICM Paris, France; 4 Medical Psychology Unit, Department of Medicine, Institute of Neuroscience, University of Barcelona, Barcelona, Catalonia Spain; 5 Institute of Biomedical Research August Pi i Sunyer (IDIBAPS), Barcelona, Catalonia Spain; 6 Cognition and Brain Plasticity Unit, Bellvitge Biomedical Research Institute (IDIBELL), L’Hospitalet de Llobregat, Barcelona, Spain; 7 Department of Cognition, Development and Educational Psychology, University of Barcelona, Barcelona, Spain; 8 Inserm U 1127, Paris, France; 9 CNRS UMR 7225, Paris, France; 10 Department of Neuroradiology, AP-HP, Hôpital Pitié-Salpêtrière, Sorbonne Université, Paris, France; 11 Fundación para la Lucha contra las Enfermedades Neurológicas de la Infancia (FLENI), Buenos Aires, Argentina; 12 Department of Physics, University of Buenos Aires, Buenos Aires, Argentina; 13 Coma Science Group, GIGA Consciousness, University of Liège, Liège, Belgium; 14 Centre du Cerveau, University Hospital of Liège, Liège, Belgium; 15 Joint International Research Unit on Consciousness, CERVO Brain Research Centre, U Laval CANADA, Québec, QC Canada; 16 International Consciousness Science Institute, Hangzhou Normal University, Hangzhou, China; 17 Department of Neurology, Christian Albrechts University, Kiel, Germany; 18 Department of Neurology and Brain Imaging Center, Goethe University, Frankfurt am Main, Germany; 19 Latin American Brain Health Institute (BrainLat), Universidad Adolfo Ibañez, Santiago, Chile; 20 Department of Psychiatry, University of Oxford, Oxford, UK; 21 Center for Music in the Brain, Department of Clinical Medicine, Aarhus University, DK, Jutland, Denmark; 22 Centre for Eudaimonia and Human Flourishing, University of Oxford, Oxford, UK; 23 Institució Catalana de la Recerca i Estudis Avancats (ICREA), Barcelona, Catalonia Spain; 24 Department of Neuropsychology, Max Planck Institute for human Cognitive and Brain Sciences, Leipzig, Germany; 25 School of Psychological Sciences, Monash University, Melbourne, Australia

**Keywords:** Computational neuroscience, Functional magnetic resonance imaging

## Abstract

Significant advances have been made by identifying the levels of synchrony of the underlying dynamics of a given brain state. This research has demonstrated that non-conscious dynamics tend to be more synchronous than in conscious states, which are more asynchronous. Here we go beyond this dichotomy to demonstrate that different brain states are underpinned by dissociable spatiotemporal dynamics. We investigated human neuroimaging data from different brain states (resting state, meditation, deep sleep and disorders of consciousness after coma). The model-free approach was based on Kuramoto’s turbulence framework using coupled oscillators. This was extended by a measure of the information cascade across spatial scales. Complementarily, the model-based approach used exhaustive in silico perturbations of whole-brain models fitted to these measures. This allowed studying of the information encoding capabilities in given brain states. Overall, this framework demonstrates that elements from turbulence theory provide excellent tools for describing and differentiating between brain states.

## Abstract (web summary)

A unifying turbulent dynamics framework using both model-free and modelbased measures of whole-brain information provides insights into brain states.

## Introduction

Fundamentally different brain states such as sleep, wakefulness, or coma all emerge from the complex dynamics of self-organised brain activity. Nevertheless, an unanswered question in modern neuroscience is how best to characterise the underlying human brain states acquired with neuroimaging^1,2^. Many challenges remain unsolved, and most importantly, there is a need to arrive at an agreed definition of brain states^2–9^. The most important feature of such a definition would help to create a mechanistic framework for characterising brain states in terms of the underlying causal mechanisms and dynamical complexity. An elegant way of assessing dynamical complexity was proposed by Massimini and colleagues who investigated the perturbation-elicited changes in global brain activity during brain states, including wakefulness, sleep, anaesthesia, and post-coma states^10–12^. They have proposed the perturbational complexity index (PCI), which captures the significant differences in brain-wide spatiotemporal propagation of external stimulation, distinguishing between different brain states^10^. Beyond basic neuroscience, a better definition and description of a brain state could offer novel avenues for translational therapeutic interventions to rebalance disrupted brain states in disease.

In a recent review, Goldman and colleagues^1^ showed that at both macroscopic and microscopic scales, unconscious brain states are dominated by synchronous activity^13–17^, while conscious states are characterised by asynchronous dynamics^15,18,19^. Equally, they propose that brain signals in unconscious and conscious states vary in their algorithmic complexity^20^, entropy^21^ and dimensionality^22^. The authors were inspired by the elegant mathematical framework of statistical physics, which provides the tools for uncovering structures of microscopic interactions underlying macroscopic properties. They propose that different brain states may emerge from the interactions between populations of neurons, similar to how different states of matter like solids and liquids emerge from interactions between populations of molecules. In other words, unconscious states are more like a solid-state, with high synchrony and low complexity, while conscious states are more like liquids, with asynchronous activity and high complexity.

This dichotomy is very useful for capturing the fundamental difference between conscious and unconscious states, especially for the microstates, where for example, deep sleep is characterised by slow waves^23^. However, the transition between scales is more subtle and crucially depends on the complex percolation across the whole brain of the synchronous and asynchronous microstates, which gives rise to mixed complex dynamical states^24^. The challenge remains to find a unifying dynamical approach, which can establish the balance between different levels of synchrony and complexity needed to distinguish between brain states.

Here, we show that different brain states are always underpinned by spatiotemporal dynamics, but the mixing across scales gives rise to dissociable dynamical characteristics, beyond simply synchronous and asynchronous signatures. We investigate this using two complementary model-free and model-based frameworks.

For the model-free framework, we profited from the advances in turbulence theory in physics^25–28^. In physical systems, starting with fluid dynamics^25–27^, turbulence has been shown to provide the optimal transmission of energy, and at the core of this transmission are the scale-free mixing properties of turbulence. Mathematically, it can be shown that energy is essentially information^29,30^. The essence of turbulence is the efficient transmission of energy/information in fluid dynamics, which was shown by Kolmogorov to be captured by elegant scale-free statistical power laws^26,27^. This shows that rather than using fine-grained Navier-Stokes equations of the billions of molecules in fluid dynamics^31^, the extremely high dimensional system of fluid dynamics can be described in a much simpler, lower-dimensional space.

Beyond this fluid dynamics approach to turbulence, Kuramoto showed that coupled oscillators can be used to capture turbulence in many other systems, suggesting that coupled oscillators could sustain optimal information transmission^28^. Specifically, within the framework of coupled oscillators, turbulence can be characterised as the variability across space and time of the *local* level of synchronisation of the coupled oscillators. In fact, this characterisation is a generalisation of the concept of metastability^32–36^, which in neuroscience has been measured as the variability across time of the *global* level of synchronisation of the whole system, commonly known as the global Kuramoto order parameter of a dynamical system.

Here, however, as in previous papers, we describe that the human brain operates in a turbulent regime^37,38^, in the sense of Kuramoto^38^, which confers important information processing advantages, including significantly enhancing the functional role of the anatomically rare long-range connections^39^. We focus on Kuramoto’s related concept of a *local* order parameter, defined as the local level of synchronisation in the system^40^. The variability of this local measure across spacetime turns out to be a sensitive and precise description of the level of turbulence. Importantly, the level of local synchronisation can be thought of as analogous to the rotational vortices found in fluid dynamics, where the size of these vortices in ‘vortex space’ defines the different scales of information processing.

In turbulence many researchers operate in such a ‘vortex space’ rather than the signal space^29,41^, which is the strategy that we also use here, noting that this is the first application of the strategy of measuring information transfer in the brain.

For the model-based framework, it has been shown that emergent collective macroscopic behaviour of brain models only depends weakly on individual neuron behaviour^42^. Here we used whole-brain modelling based on the integration of anatomy and dynamics, which can be used to accurately fit and reproduce many aspects of empirical neuroimaging data^43–46^, and specifically to capture the brain turbulent dynamics^38,47^. Over the years, there have been many different whole-brain models with varying degree of biophysical realism, from spiking networks to mean-field to oscillatory Hopf whole-brain model^43,48–52^. However, it has been shown that rather than modelling the complex spiking neuronal and mean field dynamics, very high precision fitting can be achieved by using coupled oscillators, allowing for the capture of the most important features of mesoscopic brain dynamics^49^.

Importantly, using a Hopf whole-brain model allows for in silico exhaustive perturbation of the model that can be used to assess many aspects, including the susceptibility and information encoding capability. These two measures have been defined in previous works to successfully demonstrate that the susceptibility is enhanced due to long-range connections in the brain^39^ and the information encoding capability is maximal when the brain operates in turbulent regime^38^. In other words, the model-free approach measures the naturally occurring information transmission flow, while the model-based approach allows us to measure the reactivity of the brain to external perturbations.

Furthermore, it has been shown that simply varying the global coupling in the Hopf whole-brain model produces excellent fits not only to normal resting state data but also to other brain states such as psychedelics^45^, coma, anaesthesia^53^ and sleep^45^. The most parsimonious explanation for this ability to fit multiple brain states is that the turbulence-generating Hopf model varies as function of the global coupling^37–39^. This would provide a causal mechanistic explanation of why turbulence is a sensitive and specific marker of the underlying brain state.

Overall, we hypothesised that the model-free and model-based complementary frameworks will allow us to differentiate between different brain states. We found turbulent dynamics (in the sense of Kuramoto^28^) in all the different brain states but, crucially, using the model-free framework, we were able to characterise the different information transmission across spacetime scales in resting state, meditation, deep sleep and post-coma states. Furthermore, the model-based framework showed that different information encoding capabilities^39^ characterise different brain states. Thus, according to our hypothesis, the complementary methods are able to not only significantly distinguish between different brain states but also offer a unifying dynamical framework for mechanistically describing the underlying fundamental principles.

## Results

We used model-free and model-based frameworks to explore information transmission flow in whole-brain dynamics across different brain states. Specifically, we compared brain measures on three independent resting-state fMRI datasets. The meditation dataset comprised experienced Vipassana meditators (*N* = 19) during both focused attention meditation (M) and resting state (R). The sleep dataset comprised healthy subjects during deep sleep, i.e., stage 3 (DS, *N* = 13) and resting state (R, *N* = 13) states. Finally, the disorders of consciousness (DOC) dataset were acquired in two independent research sites (Liège and Paris), comprised of healthy volunteers (R_CNT_: *N* = 49) and DOC patients diagnosed in a minimally conscious state (R_MCS_: *N* = 66) or an unresponsive wakefulness state (R_UWS_: *N* = 39).

**Fig. 1:**
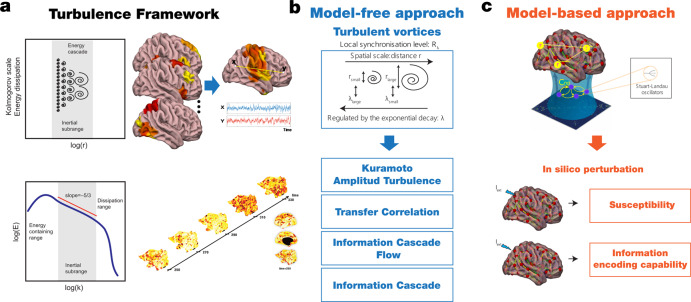
Overview of framework. **a** Turbulence in fluids is one the most common dynamical regime where the mixing motion governs (left panel). The energy cascade, i.e., how the energy travels across scale while dissipated and the statistical properties defined as power laws on the energy levels and structure functions (bottom panel) determine the turbulent behaviour of the fluid. The analogy between brain activity and Turbulence has been recently demonstrated using resting state data from a large dataset of 1,003 healthy human participants. **b** Model-free approach. The turbulent behaviour of brain activity is reflected in the similarity between the local level of synchronisation, determined by the local Kuramoto order parameter (R) at different scales (λ), and vortex with different spatial scales in fluid dynamics. The spatial scale (r) of the vortex is inversely related with the exponential decay of the local Kuramoto order parameter (λ). The turbulence regime also endows the brain with an efficient information cascade measured as the correlation of the local level of synchronisation across scales (Information Cascade Flow). The average across scales of the information cascade flow is defined as the Information cascade. The Transfer Correlation quantified as the correlation of local synchronisation across space at different scales also characterises the brain’s information processing. **c** In the Hopf whole-brain model, the dynamics of each brain area are described through a Stuart Landau non-linear oscillator. The system of local oscillators is connected through the anatomical connectivity to simulate the global dynamics, capable of reproducing statistical observables from fMRI data. We used as structural connectivity the long-range connections (LR) from human diffusion MRI measurements on top of an exponential distance rule (EDR) to fit the empirical functional connectivity as a function of the Euclidean distance (following the relation between the Kolmogorov’s second-order structure-function and the traditional FC). Using whole-brain modelling allows obtaining measures that rise from the in silico perturbative approach. We simulated external stimuli and evaluated the model’s reaction for each brain state by quantifying the susceptibility and information capability measures.

First, we applied the model-free approach to measuring information transmission flow across spacetime scales based on the recent finding demonstrating turbulence in human brain dynamics (Fig. 1a)^38^. This analysis was based on the local Kuramoto order parameter that describes the local level of synchronisation of a brain area, *n*, as a function of space, $\bar{x}$, and time, *t*, at a given scale, *λ*. The scale of the local synchronisation is defined by the parameter *λ*, which determines the size of the spatial distances where the synchronisation is evaluated, where high values of *λ* stand for short distances, and vice versa (Fig. 1b). In particular, we computed for each dataset the amplitude turbulence defined by Kuramoto as the space and time variability of the local level of synchronisation^28,40,54^ (referred here as Kuramoto amplitude turbulence), and three measures quantifying the information transmission in terms of scale, space and time correlation of the local level of synchronisation that we defined as transfer correlation, information cascade flow, and information cascade (Fig. 1 and see more details in Methods and ref. ^39^).

Second, we applied the model-based approach based on the sensitivity of these models to react to external in silico perturbations (Fig. 1c and Methods). For each brain state, we constructed a whole-brain dynamical model based on the normal form of a supercritical Hopf bifurcation coupled with the dMRI structural connectivity and the exponential distance rule (EDR). Finally, to evaluate how each model fitted reacts to external stimuli, we applied in silico perturbations by quantifying the susceptibility and information encoding capability measures.

### Model-free framework

**Fig. 2:**
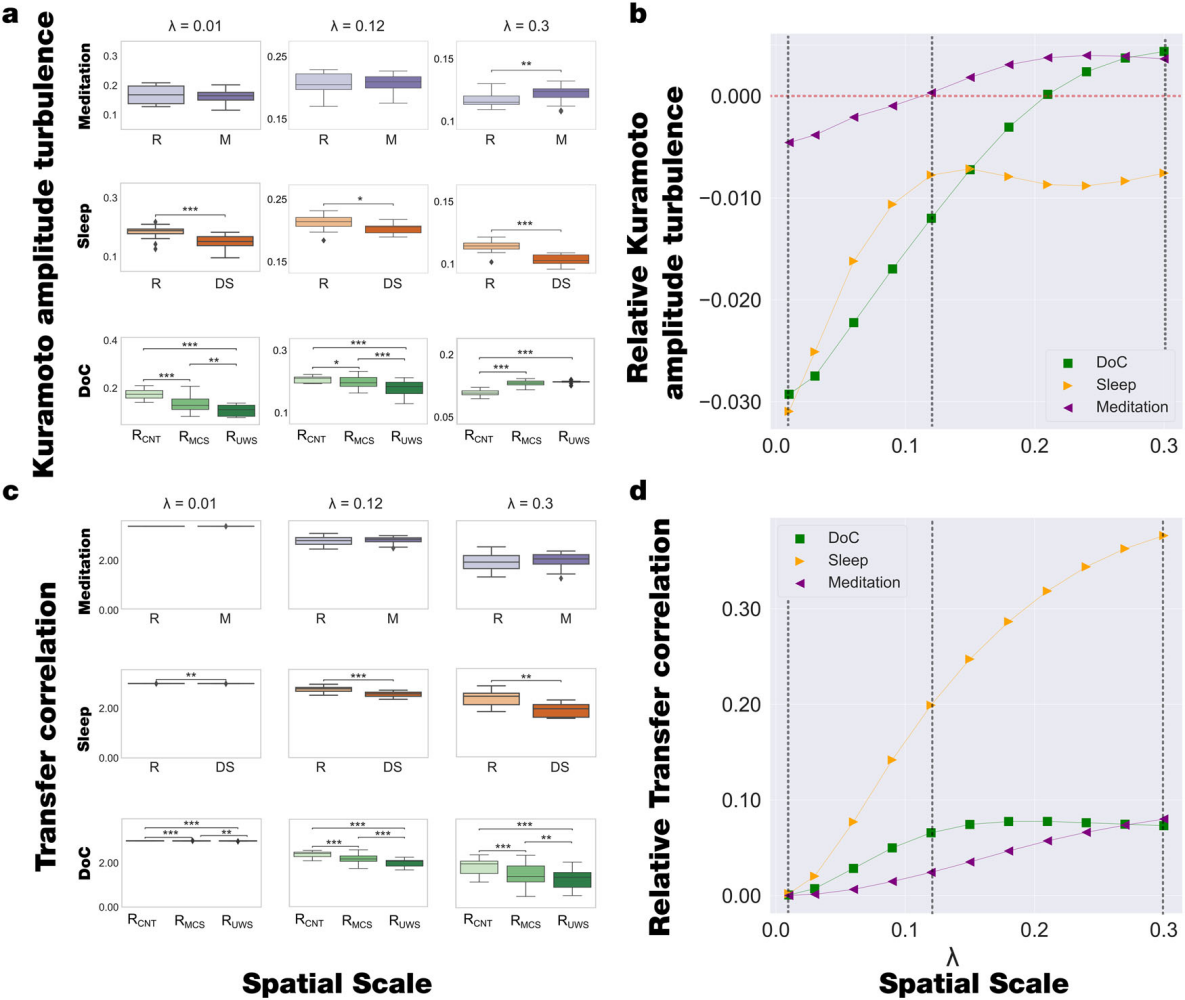
Model-free framework reveals significant differences in Kuramoto amplitude turbulence and transfer correlation in different brain states. **a** The plots show the level of Kuramoto amplitude turbulence at different spatial scales, from λ = 0.01 (100 mm) to λ = 0. 3 (3 mm) in steps of 0.03, and show the comparison between brain states for λ = 0.01, λ = 0.12 and λ = 0.3. The meditation state showed significant increases in Kuramoto amplitude turbulence compared to the resting state only on higher scales. The DS shows significantly lower Kuramoto amplitude turbulence than the resting state across all spatial scales. By contrast, the Kuramoto amplitude turbulence showed significant decreases in R_MCS_ and R_UWS_ states in lower lambda scales but significant increases in higher scales compared to R_CNT_. **b** The plots were computed as the linear fit of the mean level of Kuramoto amplitude turbulence at each scale for the three brain states for the DOC dataset (i.e., R_CNT_, R_MCS_, and R_UWS_) and two brain states for sleep and meditation datasets (i.e., W, DS, and R, M, respectively). The plots display the obtained slopes as a function of the scale. In particular, DOC showed negative slopes at lower scales and increased with the scales up to positive slopes. The sleep dataset presented negative slopes at lower scales, increased up to λ = 0.12, and a negative slope value was kept almost constant. The meditation dataset also increased with scale but presented less variability than the other datasets. Dashed vertical lines indicate the scales displayed in A and the horizontal red dashed line highlights the zero slope. **c** We computed the transfer correlation (*|A*^*λ*^|*)*, which measures how the information travels across space at different spatial scales, i.e., we show the results as a constant k - *|A*^*λ*^|, with k = 3 *|A*^*λ*^|. The meditation state presents no significant differences on any scale compared to the resting state. In contrast, the transfer correlation significantly decreased for DS and R_MCS_, R_UWS_ states compared to the resting state across all scales. **d** We performed the same computation as in panel B for the transfer correlation measure. In this case, DOC and sleep datasets presented a similar slope-scale relationship, whereas the meditation dataset presented less variability across scales. In the figure, P-values were assessed using the Wilcoxon rank-sum test and corrected for multiple comparisons, *P < 0.05, **P < 0.01 and ***P < 0.001.

We computed the information transmission flow measures on the three datasets in terms of Kuramoto amplitude turbulence and transfer correlation within the 0.008–0.08 Hz frequency range (see Methods). First, we explored the level of Kuramoto amplitude turbulence over different *λ* values, i.e., from 0.01 (~100 mm) to 0.30 (~3 mm), in steps of 0.03. This measure was defined as the standard deviation across time (*t*) and space (brain areas, *n*) of the local Kuramoto order parameter. We found that the meditation state increases Kuramoto amplitude turbulence levels in higher spatial scales, i.e., short distances in the brain, compared to the resting state. On the other hand, the deep sleep state shows lower Kuramoto amplitude turbulence levels than the resting state across all the spatial scales. Finally, the Kuramoto amplitude turbulence levels decrease for DOC patients (R_MCS_ and R_UWS_) compared to healthy controls during resting state in lower lambda values, i.e., long distances, but increases in higher lambda scales; differentiating, also, between the R_UWS_ and R_MCS_ groups. The results of Kuramoto amplitude turbulence levels in each state are displayed in Fig. 2a. We included in the supplemental material seven videos (Supplementary Videos 1 to 7) of the full spatiotemporal evolution of Kuramoto amplitude turbulence in one hemisphere across time of the full resting state of a single participant for each brain state within each dataset. Furthermore, to summarise the behaviour of the time and space information transmission measures at different scales, we quantified the Kuramoto amplitude turbulence changes at each *λ* across brain states. We computed a linear fit to the mean Kuramoto amplitude turbulence of brain states at each *λ* and obtained the slopes of the corresponding lines, which stands for Kuramoto amplitude turbulence across brain states at a specific scale. Figure 2b shows the relationship between these slopes and scales for each dataset. The meditation dataset presents similar behaviour but is less sensitive to this measure, i.e., lower variability of the slope values across scales. By contrast, the sleep dataset shows a monotonical increase of slope values from negative values at low scales up to *λ* = 0.12, where it remains almost constant for higher scales. Finally, DOC states present the same behaviour: the slopes monotonically increase from negative values at low *λ* scales towards positive values at high *λ*. This positive slopes at high λ can be associated with an increase in the short-range information transmission with a lack of a global broadcasting due to the long-range transmission diminution (negative slopes at low λ). It is noticeable that with this quantification it is possible to differentiate between datasets that involve a reduction of consciousness, i.e., despite that sleep and DOC patients present a reduction of the information processing in many scales, the behaviour across scales captures differences between sleep and DOC states.

Secondly, we explored how the information is transferred across space in terms of the time correlation between the level of local synchronisation at each scale (see Methods). This measure indicates how the information travels across space at a given spatial scale, λ. We found that the transfer correlation in the meditation state did not significantly differ across any scale compared to the resting state. By contrast, this measure significantly decreases for deep sleep and DOC states across all λ scales compared to the resting state, and interestingly, differentiating the R_MCS_ and R_UWS_ groups across all scales (Fig. 2c). Furthermore, to summarise the behaviour of transfer correlation at different scales, we quantified the changes at each λ across brain states. Conversely, the evolution of the slopes across scales for the transfer correlation presents the same behaviour across all datasets (Fig. 2d).

**Fig. 3:**
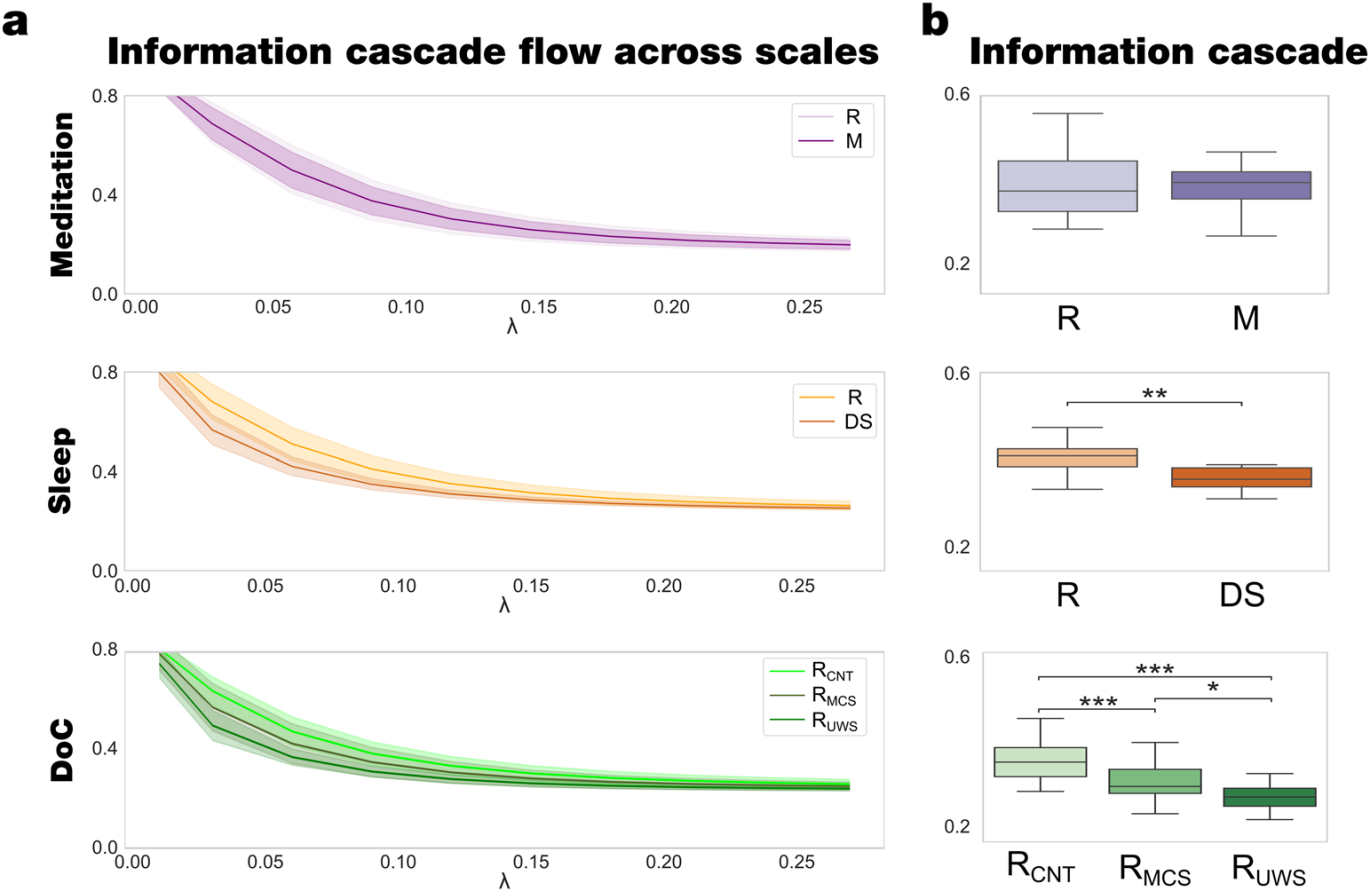
Model-free framework showed differences in information cascade flow and information cascade in different brain states. **a** The information cascade flow across scales is the predictability given by the level of synchronisation at a specific scale (λ) from the previous scale λ−∆λ (where ∆λ = 0.03 is the discretisation of scale). The meditation state presents no differences across the scales compared to the resting state, the information cascade flow significantly decreases for DS and R_MCS_, R_UWS_ states compared to the resting across all scales. **b** The information cascade, defined as the average information cascade flow, differentiates R_MCS_, R_UWS_, and DS states from the resting state, while the meditation state presents no differences. P-values were assessed using the Wilcoxon rank-sum test and corrected for multiple comparisons, *P < 0.05, **P < 0.01 and ***P < 0.001.

We measured how the information travels across scales by defining the information cascade flow, as the predictability in terms of time correlation of a given level of local synchronisation at scale *λ* from the level of local synchronisation at scale *λ*–∆*λ*, in consecutive time steps, *t* and *t* + *∆t* (where ∆λ and ∆t are the discretisation of scale and time, respectively). This is important, given that the brain is organised as a hierarchy where information flows from bottom to top in a recurrent reciprocal manner, i.e. segregated sensory information is processed first and iteratively more refined and integrated, while a global workspace at the top of the hierarchy integrates information. We found that the meditation state did not significantly changes compared to the resting state, whereas for deep sleep and DOC, the information cascade flow decreases across all scales compared to the resting state (Fig. 3a).

Finally, to summarise the information transmission’s whole behaviour across scales, we defined the information cascade as the information cascade flow average across all λ scales. We found that the information cascade in the meditation state presents no significant differences compared to the resting state. In contrast, the deep sleep and DOC states present less transfer correlation across the scales than the resting state, moreover, the information cascade clearly differentiate between R_CNT_ and R_UWS_ states (Fig. 3b).

**Fig. 4:**
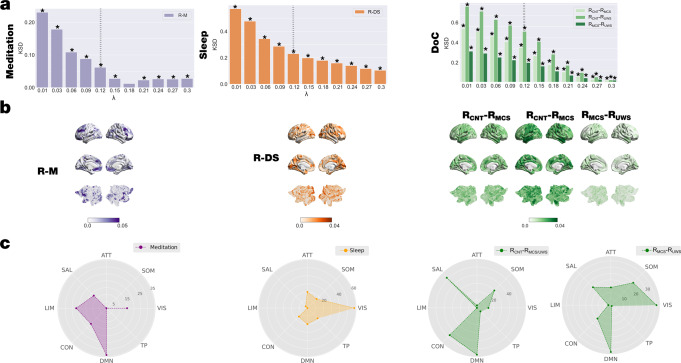
Local node-level metastability was significantly different between brain states and revealed distinct signatures of network involvement. We computed the node-level metastability as the standard deviation across time of the local Kuramoto order parameter (see Methods). **a** We performed the KSD between distributions of the node-level of metastability of each brain state within each dataset for each scale. The KSD for all datasets monotonically decreases, whereas the value of λ increases for all comparisons. **b** Render brains represent the absolute difference of the node-level metastability between each brain state for scale λ = 0.12, indicated with vertical dashed lines in panel A. We selected the top 15% quantile of absolute differences between conditions, identified the resting state networks to which they belong and quantified the number of nodes per network. **c** Radar plots represent the number of nodes on the top 15% quantile of the absolute difference by each comparison and resting-state network (CON: control; DMN: default mode; TP: temporal-parietal; VIS; visual; SOM: somatomotor; ATT: attentional; SAL: salience; LIM: limbic). The networks showing the highest differences between resting and meditation states were the limbic and default-mode networks. The comparison between deep sleep and resting state shows that nodes of the visual- and default-mode- networks present the highest difference. Finally, the comparison between *R*_*CNT*_ and DOC patients (*R*_*MCS*_ and *R*_*UWS*_) shows that the somatomotor-, salience-, control-, and default-mode- networks present the highest differences, whereas, specifically in the comparison between *R*_*MCS*_ and *R*_*UWS*_ nodes associated with the somatomotor- and control- networks present the highest differences. P-values were assessed using the Kolmogorov–Smirnov test and corrected for multiple comparisons, *P < 0.001.

To assess the regional heterogeneity of the synchronisation time variability at a given scale, we defined the node-level metastability as the standard deviation over time of the local Kuramoto order parameter for each brain state in each dataset. This measure indicates how changes the level of local synchronisation across time. We quantified this difference by computing the Kolmogorov-Smirnov distance (KSD) between the distributions of node-level metastability, where larger values mean more different distributions (see Methods). We found that the KSD for all datasets monotonically decreases between brain states across scales, whereas the value of λ increases. In other words, the KSD is maximal for lower values of λ, i.e., long distances in the brain. In particular, for DOC states, the higher KSD is found between R_CNT_ and R_UWS_ states (Fig. 4a). Furthermore, we show the absolute difference between the node-level metastability between brain states in each dataset at *λ* = 0.12 rendered onto the brain cortex (Fig. 4b).

Then, we identified the resting state networks to which they mainly belong and quantified the number of nodes per network by selecting the nodes for each comparison of the top 15% quantile. We found that differences between meditation and resting state are mainly in the limbic and default-mode networks. In contrast, the highest differences between deep sleep and resting state are observed in the nodes of the visual- and default-mode- networks. Finally, the highest differences in local synchronisation are found between controls during resting state and DOC patients (R_MCS_ and R_UWS_) in the somatomotor-, salience-, control-, and default-mode- networks. Conversely, the highest differences between R_MCS_ and R_UWS_ are observed in nodes associated with visual-, somatomotor- and default mode- networks.

### Model-based framework

**Fig. 5:**
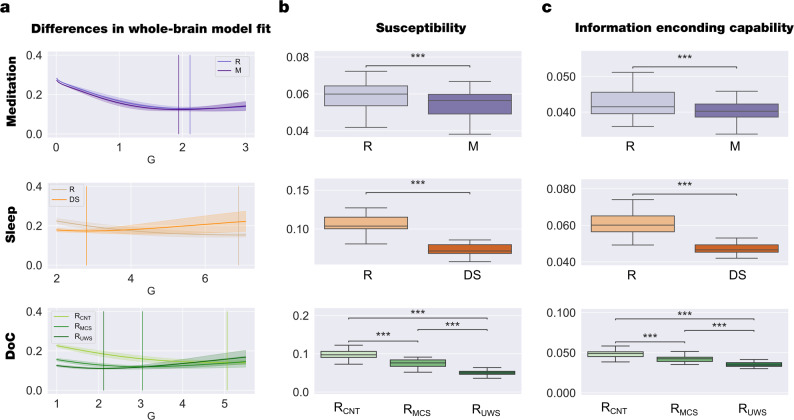
Model-based framework revealed significant perturbative differences for different brain states. **a** We show the evolution of the error of the whole-brain model FC fitting to the empirical fMRI data as a function of the global coupling strength, G. The error of the FC fitting was given by the square root of the difference between the simulated and empirical FC matrix. The optimal working point of the model was defined as the minimum value of the FC fitting, i.e., where the model shows maximal similarity to the empirical fMRI data. **b** We show the results of the susceptibility measure, which estimates how these models react to external periodical force perturbations. In all datasets, the resting state was the most susceptible to be perturbed. **c** We show the information encoding capability of the whole-brain models, which captures how different external stimulations are encoded in the dynamics. Similar to the susceptibility measure, the resting state was more susceptible to react to the perturbations. Susceptibility and information capability measures differentiated each brain state and between R_MCS_ and R_UWS_ groups. These results show that each brain state encodes the whole-brain dynamics with a particular complexity. *P*-values were assessed using the Wilcoxon rank-sum test and corrected for multiple comparisons; ***P < 0.001.

For each brain state, we built a Hopf whole-brain model of coupled dynamical oscillators in an anatomical brain architecture coupling the exponential distance rule (EDR) and the dMRI matrix fitted to the empirical functional data (see more details in Methods). In particular, we exhaustively varied *G* from 0 to 7 in 0.1 steps and for each *G* value we repeated 100 simulations for each brain state with the same TR and time duration as the empirical data. Then, we computed the fitting of the functional connectivity as the Euclidean distance between the empirical and simulated functional connectivity (FC) as a function of distance (*r)* within the inertial subrange (see Methods). The optimal working point of each model is determined as the minimum of the fitting level (vertical lines in Fig. 5a). We used the respective minima of each condition as the basis of the following perturbative in silico investigations. The *G* values obtained for meditation, deep sleep, and DOC are lower than those obtained for the resting state. This result can be interpreted as reducing the coupling between areas to represent the global brain dynamics.

Furthermore, we study how the system reacts to external perturbations by perturbing each model at its optimal working point and computing the model-based measures. Specifically, we defined the susceptibility as the ability of a system to be perturbed, and we estimated by measuring the perturbed and non-perturbed modulus of the local Kuramoto order parameter (${\widetilde{R}}_{{{{{{{\rm{\lambda }}}}}}}_{s}}\left(\bar{x},t\right)$ and ${R}_{{{{{{{\rm{\lambda }}}}}}}_{s}}\left(\bar{x},t\right)$, respectively). The perturbation consisted of applying an external periodical force equally for all brain regions. This stimulus was represented as an external additive periodic forcing term, given by *F*_*j*_ = *F*_0*j*_ cos(ω_0_ *t*) + *iF*_0*j*_ sin(ω_0_*t*) with *F*_0j_ = 5 × 10^−4^, in the corresponding real and imaginary part of the node j equation (Eqs. 10 and 11 see Methods), with frequency ω_0_ equal to the average across node of the empirical node frequency (Fig. 1c). Finally, we computed the susceptibility as the difference between the perturbed and non-perturbed cases averaged across time, trials, and space. Note that we here define susceptibility as the ability of the system to be externally perturbed, in the same sense found in electromagnetism, where the ‘magnetic susceptibility’ is determined as the magnetisation of the material as the result of an external field. This general framework was adapted for coupled oscillators by Hiroaki Daido, who defines the susceptibility of a large population of coupled oscillators as the variation of the Kuramoto order parameter under external perturbation^55^. This measure was previously used to demonstrate that the long-range connections enhance the brain responsiveness to external stimulus^39^ and also increases is turbulent regime^38^. Here we found that the susceptibility decreases for meditation, deep sleep, and DOC compared to the resting state (Fig. 5b).

Similarly, we computed the information encoding capability (as an extension of the susceptibility) to study how external perturbations are encoded in brain dynamics. This measure was defined as the standard deviation across trials of the difference between the perturbed and unperturbed mean of the modulus of the local Kuramoto order parameter across time, averaged across space. We found that, compared to the resting state, the information encoding capability also decreases for meditation, deep sleep, and DOC (Fig. 5c). To investigate the link between Information encoding capability and complexity well-establish measure, we computed the normalised Lempel-Ziv complexity^56^ (LZC) as is described in Casali et al.^10^ for each brain state within each data set when it is externally perturbed. We found that the LZC behaves similarly to the Information encoding capability measure but is less sensitive to discriminate between them (see Supplementary Fig. 1).

We replicated the results by randomly changing the bifurcation parameter of each brain area, *a*_*n*_, within the range [−0.02:0] (see Methods). As shown in Supplementary Fig. 2, we found that the response is the same for both perturbative approaches.

Overall, both perturbative measures show that the capability to react to in silico perturbations decreases for meditation, deep sleep, and DOC compared to the resting state.

## Discussion

We were able to significantly distinguish between different brain states based on a unifying framework for defining and measuring the spatiotemporal variability of local synchronisation and information transfer across scales. This research is based on Kuramoto’s important research for extending the concept of turbulence in the context of coupled oscillators^28^ (for other frameworks used to study turbulence, see^25–27,29^). Using Kuramoto’s insight, we have previously shown turbulence-like dynamics in the healthy human brain^37–39^. Here we extended these results by using model-free and model-based frameworks to demonstrate that different brain states exhibit different levels of such turbulent-like dynamics and information transfer across scales. In turbulence, such local level of synchronisation across spatial scales is usually called ‘vortex space’. Our model-free framework was able to show the role of information cascade in ‘vortex space’ as a distinguishing feature between brain states (resting state, meditation, deep sleep, R_MCS_, and R_UWS_) as measured by fMRI neuroimaging. As such our results demonstrated that these brain states exhibit significant differences in information cascade across different scales at both the spatial and temporal domains. Equally, our model-based approach fitted a whole-brain model to the empirical data, which allowed us to exhaustively perturb the system to demonstrate differences in susceptibility and information encoding capabilities between different brain states. The results showed that when inducing a shift in the intrinsic local dynamics of brain areas, the brain responds to the external perturbations less sensitively as the conscious awareness diminishes.

This framework captures the differences in percolation between scales across the whole brain of the different levels of synchrony and complexity associated with different brain states. At the mesoscopic level, the result of this percolation and mixing across scales is always reflected in the brain dynamics determined by the spatiotemporal variability of local synchronisation.

Overall, the proposed unifying framework reconciles the balance between different levels of synchrony and complexity of large population of coupled oscillators for describing and differentiating between brain states. Importantly, both model-free and model-based measures successfully differentiate the minimally conscious state (R_MCS_) and unresponsive wakefulness syndrome (R_UWS_) groups.

Previously, it has been shown that the information processing associated with rare long-range (LR) connections is significantly enhanced in the resting state of healthy awake participants^39^. When reducing the level of spatiotemporal variability of local synchronisation, in what we call the turbulent regime, for a model with LR connections, this resulted in a reduced level of long-range information transmission. While we were not explicitly testing a model with and without LR connections, we found that the evolution of Kuramoto amplitude turbulence and information cascade at different spatial scales is significantly different between different brain states. In fact, Fig. 2a and b show that compared to a group of healthy controls, the DOC groups exhibited lower levels of turbulence at higher spatial scales (i.e., lower λ and larger distances) but higher levels of turbulence at lower spatial scales. This dramatic reduction of long-range information transmission in R_UWS_ and R_MCS_ patients (see relevant boxplot for λ = 0.01 in Fig. 2a) could be a defining feature of the reduction of conscious awareness in these patients. This results are consistent with EEG evidence showing that noncommunicative patients have lower global information sharing^57^, and decreased brain complexity^58,59^. This also aligns with the global neuronal workspace theory that postulates that the long-distance connexions globally broadcast the information for different processor brain-wide and this lack of spatially bounded information processing is associated with conscious access^60^.

In healthy participants, deep sleep was characterised by lower Kuramoto amplitude turbulence across all spatial scales, demonstrating a reduction in information processing over both short and long distances^61,62^. In contrast, in highly trained meditators, the meditation state presented higher Kuramoto amplitude turbulence only at lower spatial scales (large λ and short distances), suggesting that meditation is a state showing an alteration rather than a reduction of consciousness. Overall, the results demonstrate that each brain state exhibits different turbulent dynamic patterns across spatial scales, allowing us to characterise the brain states based on fluctuations of their underlying information processing. Interestingly, this also allowed us to differentiate between deep sleep and DOC states, thus unveiling specific and unique features of turbulent dynamics underlying low-level states of consciousness, going beyond a simple dichotomy of synchrony and asynchrony.

The results also gave new insights into information processing across scales changes with brain state. Working in ‘vortex space’, we quantified three different measures of information transfer, information cascade flow and information cascade for each brain state. Figure 2c and d show that information transfer increased significantly with the spatial scale between normal resting state and the level of awareness in the other brain states (meditation, deep sleep and DOC). This result clearly demonstrates that the measure of information transfer indexes conscious awareness. Interestingly, while this measure increases with the distance between resting and meditation, this difference is not statistically significant. This suggests that meditation is more similar to the resting state but that there are important significant differences which can be revealed by the other information transmission measures.

The information cascade flow monotonically decreased with shorter distances (the increase in spatial scale λ) for all brain states (shown in Fig. 3a). This measure also discriminated *between* conditions within each dataset, i.e., showing lower values for DOC patients than control participants and in the deep sleep stage compared to the resting state in the same participants. The information cascade (i.e., the average of the information flow across scales) was lower in low-levels states of awareness (deep sleep, R_MCS_ and R_UWS_) than in normal resting state (shown in Fig. 3b). Overall, this demonstrates that the information transmission is altered with conscious access and that this is captured with the global information processing measures of information transmission, information cascade flow and information cascade.

The framework also allowed us to identify local brain regions involved in controlling the turbulent dynamics of different brain states. In particular, we defined a ‘local node-level metastability’ measure as the regional level of the variability of local synchronisation (see Methods). This measure was able to significantly differentiate between different brain states at different spatial scales. Yet, the node-level metastability for higher *λ* values, i.e., shorter distances in the brain, was less sensitive in discriminating between brain states (Fig. 4a).

Importantly, this node-level of description allowed us to capture the different signatures of the whole-brain dynamics that changed between brain states. As shown by the renderings in Fig. 4b (at *λ* = 0.12) and quantified at the network level in Fig. 4c, we found that brain regions belonging to the somatomotor, salience, control, and default-mode networks present the most critical differences in DOC states, with a more substantial decrease in the R_UWS_ than in the R_MCS_ condition, corresponding to lower levels of conscious awareness.

Specifically, we found the highest difference between R_UWS_ and R_MCS_ in brain regions belonging to default mode-, visual- and somatomotor- networks, which is consistent with previous studies in DOC patients^63–66^. We also found that changes in regions in visual- and default-mode- networks indexed differences between deep sleep and wakeful resting, consistent with other studies of the human wake-sleep cycle^67–69^. In contrast, comparing meditation with resting state in expert meditators revealed regions in limbic- and default-mode- networks, similar to other findings in meditation^70–74^.

Please note that the current study is based on human brain fMRI data. Thus, the time and spatial scales analysed here are restricted to the order of millimetres and seconds (low frequencies), respectively. Complementary to this approach, it would be of considerable interest to extend this analysis at different scales by considering different neuroimaging recording modalities capable of representing a much broader range of frequencies, such as Electrocorticography (ECoG), magnetoencephalography/ electroencephalography (MEG/EEG) and circuit level local field potentials.

Given the exciting results of directly perturbing the brain revealed by the pioneering studies of Massimini and colleagues^10–12^, we also wanted to explore the causal mechanistic underpinnings of the differences between brain states and ensuing reactivity to external perturbations. To this end, we modelled the empirical fMRI data using Hopf whole-brain models^38,45,51,68^. The question of what level of abstraction to use in the whole-brain model is the focus of much ongoing research. Over the years, there have been many different whole-brain models with varying degree of biophysical realism, from spiking networks to mean-field to oscillatory Hopf models^43,48–52^. The conclusion that we have drawn from this work is that currently, the Hopf model creates the best fitting for fMRI BOLD data^49^ with a high level of simplicity, which implies less computational cost but being cautious on the biological interpretations given the level of the abstraction of the model.

We found that the optimal working point of the Hopf whole-brain models for all brain states shifted to a lower global coupling factor compared to the resting state (see Fig. 5a). The global coupling parameter, *G*, represents the conductivity of the fibre densities among brain regions given by the underlying structural connectivity, which is assumed, for simplicity, to be equal across the brain^49,75^. Importantly, previous research showed that the optimal values of *G* and *a* are related by a monotonic function, so that fixing *a* before model fitting preserves the differences in the coupling strength parameter between states^45^. Thus, a higher coupling, *G*, allows the propagation of information among brain areas indirectly connected, enhancing the transmission of information across the whole network and vice versa^53^. Overall, this drastic shift toward a lower coupling indicates sub-critical behaviour suggestive of a change in the dynamical complexity underlying the brain state^75^.

In other words, simply varying the global coupling, G, in the Hopf model have allowed us to obtain an excellent fit for different brain states such as psychedelics^76^, DOC^53,77^, anaesthesia^53^ and sleep^45^. One can of course add more parameters to the Hopf model, such effective connectivity which creates an even better fit to the empirical data^78^. One can also use more sophisticated biophysical grounded models that provide a set of parameters with a different biological interpretation that could provide new insight into the differences between brain states. Nevertheless, utilising Occam’s razor, we went for the minimal Hopf model that can reproduce the differences in brain states.

Using the model-based framework, crucially, we were able to perturb each brain model at its optimal working point to investigate the induced whole-brain dynamics changes caused by the external in silico perturbations in order to obtain complementary measures of information encoding in different brain states. Specifically, our external manipulation consisted of a shift towards the bifurcation point of the intrinsic local dynamics of brain areas. We found that the resting state showed significantly higher susceptibility and information encoding capability than in the pairwise comparison in each dataset, i.e., meditation, deep sleep and DOC. The similar behaviour of both measures (susceptibility and information encoding capability) can be related to the specific features of our perturbative approach. Differences in silico protocols can be assessed to study how different brain states react to external perturbations such as shifting the local dynamics in the opposite direction, node by node perturbation^78,79^, non-sustained perturbations^47,80^ or perturbing with external strength dependent periodic force^47,77^. Notably, the perturbative approach allows for the exploration of brain responses elicited by in silico protocols which are not limited by ethical constraints of in vivo stimulations^81,82^. Furthermore, the differential sensitivity of each brain state of external perturbations could potentially serve as a specific biomarker that reveals features of their dynamical complexity.

Overall, we have presented a unifying framework that can account for the differences between brain states. The key idea is that the complex dynamics of a brain state result from the percolation across scales of previously demonstrated differences in synchrony and complexity at the microscale. These dynamics present differentiable turbulent dynamics, in terms of spatiotemporal variability of local synchronisation, which our dual model-free and model-based framework can reveal. The main finding is that turbulent dynamics across different spatial scales can distinguish between brain states. Furthermore, these differences are also found as differences in susceptibility and information encoding capability as a result of the reactivity of different external perturbations on the underlying brain state. Given the sensitivity and specificity of the results, long-term, these might help identify potential targets for patients to rebalance and regain consciousness.

## Methods

### Participants

#### Meditation

A total of 19 experienced meditators with more than 1000 hours of meditation experience were selected from a dataset previously described in Escrichs et al. (2019)^83^. Meditators were recruited from Vipassana communities of Barcelona, Catalonia, Spain (7 females, mean ± SD, 39.8 ± 10.29 years, 9,526.9 ± 8,619.8 meditation experience). Participants were asked to practice focused attention on breathing (i.e., anapanasati in language Pali). In this meditation technique, meditators focus their attention on natural breathing, and when they realize that the mind is wandering, they must refocus their attention back to natural breathing. All participants reported no history of past neurological disorder and gave written informed consent. The study was approved by the Ethics Committee of the Bellvitge University Hospital according to the Helsinki Declaration on ethical research.

#### Sleep

A total of 63 healthy subjects (36 females, mean ± SD, 23 ± 43.3 years) were selected from a dataset previously described in Tagliazucchi and Laufs^84^. On the day of the study, participants reported a wake-up time between 5:00 AM and 11:00 AM and a sleep onset time between 10:00 PM and 2:00 AM for the night before the experiment. Within half an hour of 7 PM, participants entered the scanner and were asked to relax, close their eyes, and not fight the sleep onset. Their resting state activity was measured for 52 minutes with a simultaneous combination of EEG and fMRI. According to the rules of the American Academy of Sleep Medicine^85^, the scalp potentials measured with EEG determine the classification of sleep into four stages (resting state, N1, N2 and N3 sleep). We selected 13 subjects who reached the deep sleep stage (DS, i.e., N3) and contiguous time series of at least 198 volumes. The local ethics committee approves the experimental protocol (Goethe-Universität Frankfurt, Germany, protocol number: 305/07), and written informed consent was asked to all participants before the experiment. The study was conducted according to the Helsinki Declaration on ethical research.

#### Disorders of consciousness, Paris

A total of 77 patients who were hospitalised in Paris Pitié-Salpêtrière, suffering from brain injuries, were included in this study. Clinical assessment and trained clinicians carried out the clinical assessment and Coma Recovery Scale-Revised (CRS-R) scoring to determine their state of consciousness. Patients were diagnosed with UWS if they showed arousal (opening their eyes) without any signs of awareness (never exhibiting non-reflex voluntary movements). On the other hand, patients were in a R_MCS_ if they exhibited some behaviours that could be indicative of awareness, such as visual pursuit, orientation to pain, or reproducible command following. We excluded subjects with T1 acquisition errors (*n* = 5), with high levels of motion detected (*n* = 7), registration errors (*n* = 4), and large focal brain lesions (*n* = 4). We thus included 33 patients in MCS (11 females, mean age ± SD, 47.25 ± 20.76 years), and 24 in UWS (10 females, mean age ± SD, 39.25 ± 16.30 years) and 13 healthy controls (7 females, mean age ± SD, 42.54 ± 13.64 years). This research was approved by the local ethics committee Comité de Protection des Personnes Ile de France 1 (Paris, France) under the code ‘Recherche en soins courants’ (NEURODOC protocol, n° 2013-A01385-40). The patient’s family gave their informed consent for the participation of their relative, and all investigations were conducted according to the Declaration of Helsinki and the French regulations.

#### Disorders of consciousness, Liège

A total of 35 healthy controls (14 females, mean age ± SD, 40 ± 14 years) and 48 patients with disorders of consciousness (DOC) were included in the study based on a dataset previously described in López-González et al^53^. The diagnosis was made after at least 5 CRS-R by trained clinicians. The highest diagnosis of the level of consciousness was taken as the final diagnosis, which was also confirmed with Positron Emission Tomography (PET) (i.e., patients in MCS presented a relatively preserved metabolism in the fronto-parietal network while patients with UWS had a bilateral hypometabolism in this network). We thus included 33 patients in MCS (9 females, mean age ± SD, 45 ± 16 years), and 15 in UWS (6 females, mean age ± SD, 47 ± 16 years). The Ethics Committee of the Faculty of Medicine of the University of Liege approved the study protocol. The study was conducted according to the Helsinki Declaration on ethical research. Written informed consent was obtained from controls and the patients’ legal surrogates.

### MRI data acquisition

#### Meditation

MRI images were acquired on a 3 T Siemens Trio scanner (Siemens, Erlangen, Germany) using a 32-channel receiver coil. The high-resolution T1-weighted images were acquired with 208 contiguous sagittal slices; TR/TE = 1970 ms/ 2.34 ms; inversion time (IT) = 1050 ms; flip angle = 9°; FOV = 256 mm; and isotropic voxel size 1 mm. Resting-state and meditation fMRI images were performed by a single shot gradient-echo EPI sequence with a total of 450 volumes (15 min); TR/TE = 2000 ms/29 ms; FOV = 240 mm; in-plane resolution 3 mm; 32 transversal slices with thickness = 4 mm; flip angle = 80°.

#### Sleep

MRI images were acquired on a 3-T Siemens Trio scanner (Erlangen, Germany). EEG via a cap (modified BrainCapMR, Easycap, Herrsching, Germany) was recorded continuously during fMRI acquisition (1505 volumes of T2-weighted echo planar images, TR/TE = 2080 ms/30 ms, matrix 64 × 64, voxel size 3 × 3 × 2 mm3, distance factor 50%; FOV 192 mm^2^). An optimised polysomnographic setting was employed (chin and tibial EMG, ECG, EOG recorded bipolarly [sampling rate 5 kHz, low pass filter 1 kHz] with 30 EEG channels recorded with FCz as the reference [sampling rate 5 kHz, low pass filter 250 Hz]. Pulse oxymetry and respiration were recorded via sensors from the Trio [sampling rate 50 Hz]) and MR scanner compatible devices (BrainAmp MR + , BrainAmpExG; Brain Products, Gilching, Germany), facilitating sleep scoring during fMRI acquisition.

#### Disorders of consciousness, Paris

MRI images were acquired with two different acquisition protocols. In the first protocol, MRI data of 26 patients and 13 healthy controls were acquired on a 3T General Electric Signa System. T2*-weighted whole brain resting state images were acquired with a gradient-echo EPI sequence using axial orientation (200 volumes, 48 slices, slice thickness: 3 mm, TR/TE: 2400 ms/30 ms, voxel size: 3.4375 × 3.4375 × 3.4375 mm, flip angle: 90°, FOV: 220 mm^2^). An anatomical volume was also acquired using a T1-weighted MPRAGE sequence in the same acquisition session (154 slices, slice thickness: 1.2 mm, TR/TE: 7.112 ms/3.084 ms, voxel size: 1 × 1 × 1 mm, flip angle: 15°).

In the second protocol, MRI data of 51 patients were acquired on a 3 T Siemens Skyra System. T2*-weighted whole brain resting state images were recorded with a gradient-echo EPI sequence using axial orientation (180 volumes, 62 slices, slice thickness: 2.5 mm, TR/TE: 2000 ms/30 ms, voxel size: 2 × 2 × 2 mm, flip angle: 90°, FOV: 240 mm^2^, multiband factor: 2). An anatomical volume was acquired in the same session using a T1-weighted MPRAGE sequence (208 slices, slice thickness: 1.2 mm, TR/TE: 1800 ms/2.35 ms, voxel size: 0.85 × 0.85 × 0.85 mm, flip angle: 8°).

#### Disorders of consciousness, Liège

MRI images were acquired on a Siemens 3 T Trio scanner (Siemens Inc, Munich, Germany). MRI acquisition included a gradient echo-planar imaging (EPI) sequence (32 transversal slices, 300 volumes, TR/TE = 2000 ms/30 ms, flip angle = 78°, voxel size = 3x3x3 mm, FOV = 192 mm); a structural T1 (120 transversal slices, TR = 2300 ms, voxel size = 1.0 × 1.0 × 1.2 mm, flip angle = 9°, FOV = 256 mm).

### Brain parcellation

We used the Schaefer parcellation with 1000 brain areas, based on estimation from a large dataset (*n* = 1489)^86^, to extract the time series from each subject. Furthermore, we estimated the Euclidean distances from the Schaefer parcellation in MNI space.

### Resting-state pre-processing

#### For meditation, Paris, Liège datasets

The pre-processing of resting-state data was performed using FSL (http://fsl.fmrib.ox.ac.uk/fsl) as described in our previous study^53^. In brief, resting-state fMRI was computed using MELODIC (Multivariate Exploratory Linear Optimised Decomposition into Independent Components)^87^. Steps included discarding the first five volumes, motion correction using MCFLIRT^88^, Brain Extraction Tool (BET)^89^, spatial smoothing with 5 mm FWHM Gaussian Kernel, rigid-body registration, high pass filter cutoff = 100.0 s, and single-session ICA with automatic dimensionality estimation. Then, lesion-driven artifacts (for patients) and noise components were regressed out independently for each subject using FIX (FMRIB’s ICA-based X-noiseifier)^90^. Finally, FSL tools were used to co-register the images and extract the time-series between 1000 cortical brain areas for each subject in MNI space from the Schaefer parcellation^86^.

#### For the sleep dataset

The pre-processing of resting-state data was performed using FSL (http://fsl.fmrib.ox.ac.uk/fsl). In brief, steps included discarding the first five volumes, motion correction using MCFLIRT^88^, BET^89^, spatial smoothing with 5 mm FWHM Gaussian Kernel, rigid-body registration, bandpass filtering between 0.01 − 0.1 Hz. Finally, FSL tools were used to co-register the images and extract the time-series between 1000 cortical brain areas for each subject in MNI space from the Schaefer parcellation^86^. Previous publications based on this dataset can be consulted for further details^77^.

### Probabilistic Tractography analysis

We used the Human Connectome Project (HCP) database that contains diffusion spectrum and T2-weighted neuroimaging data from 32 participants as reported in Deco and Kringelbach^38^. A complete description of the acquisition parameters for diffusion MRI (dMRI) is described in detail on the HCP website^91^. The freely Lead-DBS software package (https://www.lead-dbs.org/) provides the pre-processing described in detail in Horn et al.^92^. In brief, the data were processed by using a q-sampling imaging algorithm implemented in DSI studio (http://dsi-studio.labsolver.org). A white-matter mask was computed by segmenting the T2-weighted images and co-registering the images to the b0 image of the diffusion data using SPM12. For each HCP participant, 200,000 fibres were sampled within the white-matter mask. Fibres were transformed into MNI space using Lead-DBS Horn and Blankenburg^93^. Finally, we used the standardised methods in Lead-DBS to extract the structural connectomes from the Schaefer 1000 parcellation^86^.

### Model-free framework

#### Kuramoto Local order parameter

The amplitude turbulence, ${R}_{\lambda }\left(\bar{x},t\right)$, is defined as the modulus of the Kuramoto local order parameter for a given brain area as a function of time:

1 $${R}_{{{\lambda}}}(\bar{x},t){e}^{i{{{\vartheta }}}_{{{\lambda}}}\left(\bar{x},t\right)}={{{{{\rm{k}}}}}}\int _{-\infty}^{{{\infty}}}d\bar{x}^{\prime} {G}_{{{\lambda }}}\left(\bar{x}-{\bar{x}}^{{\prime} }\right){{{{{{\rm{e}}}}}}}^{{{{{{\rm{i}}}}}}{{\varphi }}\left({\bar{x}}^{{\prime} },{{{{{\rm{t}}}}}}\right)}$$

where *G*_*λ*_ is the local weighting kernel ${G}_{{{{{{\rm{\lambda }}}}}}}\left(\bar{x}\right)={e}^{-{{{{{\rm{\lambda }}}}}}\left|\bar{x}\right|}$, *λ* is the spatial scaling and ${{{{{\rm{\varphi }}}}}}\left(\bar{x},{{{{{\rm{t}}}}}}\right)$ are the phases of the spatiotemporal data and k is the normalisation factor ${[{\int }_{-\infty }^{\infty }d\bar{x}^{\prime} {G}_{\lambda }(\bar{x}-{\bar{x}}^{{\prime} })]}^{-1}$. The empirical instantaneous phases were computed applying the Hilbert transform to the narrowband of 0.008–0.08 Hz filtered BOLD signals individually. This frequency range was chosen because it has been shown that when mapped to the grey matter, this band contains more reliable and functionally relevant information compared to other frequency bands, and is less affected by noise.^94^

Thus, *R*_*λ*_ defines local levels of synchronisation at a given scale, *λ*, as function of space, $\bar{x}$, and time, *t*. This measure captures what we call *brain vortex space, R*_*λ*_, over time, inspired by the rotational vortices found in fluid dynamics, but of course not identical.

#### Amplitude turbulence

The level of amplitude turbulence, *D*_*λ*_, is defined as the standard deviation across time and space of the modulus of local Kuramoto order parameter (R):

2 $${D}_{\lambda }=\sqrt{{\left\langle {{R}_{\lambda }}^{2}\right\rangle }_{x,t}-{\left\langle {R}_{\lambda }\right\rangle }_{x,t}^{2}}$$

where the brackets ${\left\langle \right\rangle }_{x,t}$ denotes averages across time and space.

#### Information cascade flow and Information cascade

The information cascade flow indicates how travels the information from a given scale (*λ*) to a lower scale (*λ* − ∆*λ*, where ∆*λ* is a scale step) in consecutive time steps (*t* and *t* + ∆*t*). In this sense, the information cascade flow measures the information transfer across scales computed as the time correlation between the Kuramoto local order parameter in two consecutive scales and times:

3 $${{{{{\mathscr{F}}}}}}\left(\lambda \right)={\left\langle {{corr}}_{t}({R}_{\lambda }\left(\bar{x},t+\Delta t\right),{R}_{\lambda -\Delta \lambda }\left(\bar{x},t\right))\right\rangle }_{\bar{x}}$$

where the brackets ${\left\langle \right\rangle }_{x,t}$ denotes averages across time and space. Then, the information cascade is obtained by averaging the information cascade flow across scales *λ*, which captures the whole behaviour of the information processing across scales (Fig. 1a, middle panel).

#### Transfer Correlation

The spatial Transfer Correlation indicates how the information travels across space at a specific scale, *λ*. This measurement is computed as the slope of a linear fitting in the log-log scale of the time correlation between the Kuramoto local order parameter of two brain areas at the same scale as a function of its Euclidean distance (*r*) within the inertial subrange (Fig. 1a, right panel).

4 $${\log }\left({cor}{r}_{t}\left({R}_{n}^{\lambda },{R}_{p}^{\lambda }\right)\left(r\right)\right)={A}^{\lambda }* {\log }\left(r\right)+{B}^{\lambda }$$

Essentially, A^λ^ and B^λ^ are the fitting parameters for each scale (λ), where *r* is the spatial distance in brain. The negative slope (A^λ^) stands for the transfer in the spatial direction *r* of the information in terms of time correlation of the local level of synchronisation. In this sense, when the slope is steeper, the information travels across shorter distances; while a flatter slope indicates that the information is transferred across longer distances. Thus, we define the negative slope as the *spatial transfer correlation*. Please note that in order to represent longer distances of information transmission with higher positive values, we present the results panels of Fig. 2c as a constant value minus |A^λ^|.

#### Local node-level metastability

We define the ‘local node-level metastability’ as the brain region variability of the local synchronisation, measured as the standard deviation across time of the local Kuramoto order parameter:

5 $${{{{{\rm{NLM}}}}}}\left({{{{{\rm{n}}}}}},{{{{{\rm{\lambda }}}}}}\right)=\sqrt{{\left\langle {R}_{n}^{\lambda }{\left(t\right)}^{2}\right\rangle }_{t}-{\left\langle {R}_{n}^{\lambda }\left(t\right)\right\rangle }_{t}^{2}}$$

where the brackets < >_*t*_ represent average values across time points.

Here, we used the discrete version of the node-level Kuramoto order parameter, with modulus *R* and phase *ν*, representing a spatial average of the complex phase factor of the local oscillators weighted by the coupling computed in the following way:

6 $${R}_{n}^{\lambda }\left(t\right){e}^{{{{{{\rm{i}}}}}}{\nu }_{{{{{{\rm{n}}}}}}}({{{{{\rm{t}}}}}})}=\mathop{\sum }\limits_{p}\left[\frac{{{{{{{\rm{C}}}}}}}_{{{{{{\rm{np}}}}}}}^{\lambda }}{{\sum }_{{{\mbox{q}}}}{{{{{{\rm{C}}}}}}}_{{{{{{\rm{nq}}}}}}}^{\lambda }}\right]{{{\mbox{e}}}}^{{{{\mbox{i}}}{{{{{\rm{\varphi }}}}}}}_{{{\mbox{p}}}}\left({{\mbox{t}}}\right)}$$

where *ϕ*_*p*_(*t*) are the phases of the spatiotemporal data and ${{{{{{\rm{C}}}}}}}_{{{{{{\rm{nq}}}}}}}^{{{{{{\rm{\lambda }}}}}}}$ is the local weighting kernel between node *n* and *p*, and *λ* defines the spatial scaling:

7 $${C}_{{np}}={e}^{-\lambda \left(r\left(n,p\right)\right)}$$

where *r*(*n, q*) is the Euclidean distance between the brain areas *n* and *p* in MNI space.

To compare the node-level metastability statistics, we collected the 1000 nodes values for all participants in each condition and generated the distributions. Then, we compared across states the distributions using the Kolmogorov-Smirnov distance between them. The Kolmogorov–Smirnov distance quantifies the maximal difference between the cumulative distribution functions of the two samples, where larger values stand for more significant differences between both distributions.

### Model-based framework

We constructed whole-brain dynamical models based on the normal form of a supercritical Hopf bifurcation (also known as Stuart-Landau)^49^. This type of bifurcation can change the qualitative nature of the solutions from a limit cycle that yields self-sustained oscillations towards a stable fixed point in phase space. This whole-brain computational model is characterised by a series of model parameters that rules the global dynamical behaviour. One of them is the multiplicative factor, *G*, representing the global conductivity of the fibres scaling the structural connectivity between brain areas, which is assumed to be equal across the brain^49,75^. The other relevant parameters are the local bifurcation parameter (*a*_*j*_), which rules the dynamical behaviour of each area between noise-induced (*a* < 0), self-sustained oscillations (*a* > 0) or a critical behaviour between both (*a* ~ 0) (Fig. 1c). We optimised the model parameters to better fit the empirical functional connectivity as a function of the distance, *r*, within the inertial subrange. The models consisted of 1000 cortical brain areas from the resting-state atlas mentioned above. The underlying anatomical matrix *C*_*np*_ was added to link the brain structure and functional dynamics and was obtained by measuring the exponential distance rule as defined in Eq. (7). The local dynamics of each brain area was described by the normal form of a supercritical Hopf bifurcation, which emulates the dynamics for each brain area from noisy to oscillatory dynamics as follows:

8 $$\frac{{{{{{\rm{d}}}}}}{{{{{{\rm{x}}}}}}}_{{{{{{\rm{n}}}}}}}}{{{{{{\rm{dt}}}}}}}={{{{{{\rm{a}}}}}}}_{{{{{{\rm{n}}}}}}}{{{{{{\rm{x}}}}}}}_{{{{{{\rm{n}}}}}}}-\left[{{{{{{\rm{x}}}}}}}_{{{{{{\rm{n}}}}}}}^{2}+{{{{{{\rm{y}}}}}}}_{{{{{{\rm{n}}}}}}}^{2}\right]{{{{{{\rm{x}}}}}}}_{{{{{{\rm{n}}}}}}}-{{{{{{\rm{\omega }}}}}}}_{{{{{{\rm{n}}}}}}}{{{{{{\rm{y}}}}}}}_{{{{{{\rm{n}}}}}}}+{{{{{\rm{\nu }}}}}}{{{{{{\rm{\eta }}}}}}}_{{{{{{\rm{n}}}}}}}\left({{{{{\rm{t}}}}}}\right)$$

9 $$\frac{{{{{{\rm{d}}}}}}{{{{{{\rm{y}}}}}}}_{{{{{{\rm{n}}}}}}}}{{{{{{\rm{dt}}}}}}}={{{{{{\rm{a}}}}}}}_{{{{{{\rm{n}}}}}}}{{{{{{\rm{y}}}}}}}_{{{{{{\rm{n}}}}}}}-\left[{{{{{{\rm{x}}}}}}}_{{{{{{\rm{n}}}}}}}^{2}+{{{{{{\rm{y}}}}}}}_{{{{{{\rm{n}}}}}}}^{2}\right]{{{{{{\rm{y}}}}}}}_{{{{{{\rm{n}}}}}}}+{{{{{{\rm{\omega }}}}}}}_{{{{{{\rm{n}}}}}}}{{{{{{\rm{x}}}}}}}_{{{{{{\rm{n}}}}}}}+\nu {{{{{{\rm{\eta }}}}}}}_{n}\left({{{{{\rm{t}}}}}}\right)$$

where *η*_*n*_(*t*) is additive Gaussian noise with standard deviation *ν* = 0.01. This normal form has a supercritical bifurcation at *a*_*n*_ = 0, such that for *a*_*n*_ > 0, the system is in a stable limit cycle oscillation with frequency *f*_*n*_ = *ω*_*n*_/2*π*, whereas for *a*_*n*_ < 0, the local dynamics are in a stable point (i.e., noisy state). The frequency *ω*_*n*_ of each brain area was estimated from the empirical fMRI data as the peak of the power spectrum.

Finally, the whole-brain dynamics was defined by the following set of coupled equations:

10 $$\frac{{{{{{\rm{d}}}}}}{{{{{{\rm{x}}}}}}}_{{{{{{\rm{n}}}}}}}}{{{{{{\rm{dt}}}}}}}={{{{{{\rm{a}}}}}}}_{{{{{{\rm{n}}}}}}}{{{{{{\rm{x}}}}}}}_{{{{{{\rm{n}}}}}}}-\left[{{{{{{\rm{x}}}}}}}_{{{{{{\rm{n}}}}}}}^{2}+{{{{{{\rm{y}}}}}}}_{{{{{{\rm{n}}}}}}}^{2}\right]{{{{{{\rm{x}}}}}}}_{{{{{{\rm{n}}}}}}}-{{{{{{\rm{\omega }}}}}}}_{{{{{{\rm{n}}}}}}}{{{{{{\rm{y}}}}}}}_{{{{{{\rm{n}}}}}}}+{{{{{\rm{G}}}}}}\mathop{\sum }\limits_{{{{{{\rm{p}}}}}}=1}^{{{{{{\rm{N}}}}}}}{{{{{{\rm{C}}}}}}}_{{{{{{\rm{np}}}}}}}\left({{{{{{\rm{x}}}}}}}_{{{{{{\rm{p}}}}}}}\left({{{{{\rm{t}}}}}}\right)-{{{{{{\rm{x}}}}}}}_{{{{{{\rm{n}}}}}}}\right)+{{{{{\rm{\nu }}}}}}{{{{{{\rm{\eta }}}}}}}_{{{{{{\rm{n}}}}}}}\left({{{{{\rm{t}}}}}}\right)$$

11 $$\frac{{{{{{\rm{d}}}}}}{{{{{{\rm{y}}}}}}}_{{{{{{\rm{n}}}}}}}}{{{{{{\rm{dt}}}}}}}={{{{{{\rm{a}}}}}}}_{{{{{{\rm{n}}}}}}}{{{{{{\rm{y}}}}}}}_{{{{{{\rm{n}}}}}}}-\left[{{{{{{\rm{x}}}}}}}_{{{{{{\rm{n}}}}}}}^{2}+{{{{{{\rm{y}}}}}}}_{{{{{{\rm{n}}}}}}}^{2}\right]{{{{{{\rm{y}}}}}}}_{{{{{{\rm{n}}}}}}}+{{{{{{\rm{\omega }}}}}}}_{{{{{{\rm{n}}}}}}}{{{{{{\rm{x}}}}}}}_{{{{{{\rm{n}}}}}}}+{{{{{\rm{G}}}}}}\mathop{\sum }\limits_{{{{{{\rm{p}}}}}}=1}^{{{{{{\rm{N}}}}}}}{{{{{{\rm{C}}}}}}}_{{{{{{\rm{np}}}}}}}\left({{{{{{\rm{y}}}}}}}_{{{{{{\rm{p}}}}}}}({{{{{\rm{t}}}}}})-{{{{{{\rm{y}}}}}}}_{{{{{{\rm{p}}}}}}}\right)+{{{{{\rm{\nu }}}}}}{{{{{{\rm{\eta }}}}}}}_{{{{{{\rm{n}}}}}}}\left({{{{{\rm{t}}}}}}\right)$$

Where the global coupling factor G, scaled equally for each brain area, represents the input received in region *n* from every other region *p*.

For the functional connectivity fitting the Kolmogorov’s structure-function of a variable *u* was applied to the BOLD signal of the data. This measure is based on the functional correlations between each pair of brain areas with equal Euclidean distance and was defined as:

12 $${{{{{\rm{S}}}}}}\left({{{{{\rm{r}}}}}}\right)={\left\langle {\left(u\left(\bar{x}+r\right)-u\left(\bar{x}\right)\right)}^{2}\right\rangle }_{x,t}=2\left[{FC}\left(0\right)-{FC}\left({{{{{\rm{r}}}}}}\right)\right]$$

where *FC*(*r*) is the spatial correlations of two points separated by a Euclidean distance *r*, which is given by:

13 $${FC}\left(r\right)={\left\langle u\left(\bar{x}+r\right)u\left(\bar{x}\right)\right\rangle }_{\bar{x},t}$$

where the symbol ${\left\langle \right\rangle }_{x,t}$ refers to the average across the spatial location *x* of the brain areas and time. Thus, the structure functions characterise the evolution of the functional connectivity (FC) as a function of the Euclidean distance between equally distant nodes, which is different from the usual definition of FC that does not include distance. We then compute the fitting as the Euclidean distance between simulated and empirical FC(r) within the inertial range as defined in Deco et al.^38^.

The main implementation consists of an external perturbation represented as an external additive periodical forcing term in the Hopf brain model for each brain states as follows:

14 $$\frac{{{{{{\rm{d}}}}}}{{{{{{\rm{x}}}}}}}_{{{{{{\rm{n}}}}}}}}{{{{{{\rm{dt}}}}}}}=	\, {{{{{{\rm{a}}}}}}}_{{{{{{\rm{n}}}}}}}{{{{{{\rm{x}}}}}}}_{{{{{{\rm{n}}}}}}}-\left[{{{{{{\rm{x}}}}}}}_{{{{{{\rm{n}}}}}}}^{2}+{{{{{{\rm{y}}}}}}}_{{{{{{\rm{n}}}}}}}^{2}\right]{{{{{{\rm{x}}}}}}}_{{{{{{\rm{n}}}}}}}-{{{\omega }}}_{{{{{{\rm{n}}}}}}}{{{{{{\rm{y}}}}}}}_{{{{{{\rm{n}}}}}}}+{{{{{\rm{G}}}}}}\mathop{\sum }\limits_{{{{{{\rm{p}}}}}}=1}^{{{{{{\rm{N}}}}}}}{{{{{{\rm{C}}}}}}}_{{{{{{\rm{np}}}}}}}\left({{{{{{\rm{x}}}}}}}_{{{{{{\rm{p}}}}}}}\left({{{{{\rm{t}}}}}}\right)-{{{{{{\rm{x}}}}}}}_{{{{{{\rm{n}}}}}}}\right)\\ 	 +{F}_{0j}{{\cos }}\left({\omega }_{0j}t\right)+{v\eta }_{n}(t)$$

15 $$\frac{{{{{{\rm{d}}}}}}{{{{{{\rm{y}}}}}}}_{{{{{{\rm{n}}}}}}}}{{{{{{\rm{dt}}}}}}}= 	\,{{{{{{\rm{a}}}}}}}_{{{{{{\rm{n}}}}}}}{{{{{{\rm{y}}}}}}}_{{{{{{\rm{n}}}}}}}-\left[{{{{{{\rm{x}}}}}}}_{{{{{{\rm{n}}}}}}}^{2}+{{{{{{\rm{y}}}}}}}_{{{{{{\rm{n}}}}}}}^{2}\right]{{{{{{\rm{y}}}}}}}_{{{{{{\rm{n}}}}}}}+{{{\omega }}}_{{{{{{\rm{n}}}}}}}{{{{{{\rm{x}}}}}}}_{{{{{{\rm{n}}}}}}}+{{{{{\rm{G}}}}}}\mathop{\sum }\limits_{{{{{{\rm{p}}}}}}=1}^{{{{{{\rm{N}}}}}}}{{{{{{\rm{C}}}}}}}_{{{{{{\rm{np}}}}}}}\left({{{{{{\rm{y}}}}}}}_{{{{{{\rm{p}}}}}}}\left({{{{{\rm{t}}}}}}\right)-{{{{{{\rm{y}}}}}}}_{{{{{{\rm{p}}}}}}}\right)\\ 	 +{F}_{0j}{{\sin }}\left({\omega }_{0j}t\right)+{v\eta }_{n}(t)$$

where ω_0_ average empirical node frequency equal to all the nodes. The strength of the external periodical force was fixed at *F*_0j_ = 5 × 10^−4^ equally for all nodes based on previous results presented in Perl et al.^47^.

In the alternative implementation we perturb the Hopf whole-brain model at each brain state by randomly changing the local bifurcation parameter, a_n_, in the range [−0.02:0]. Note that this perturbation is carefully defined to keep the dynamical scenario in the subcritical regime of each oscillator. For further detail in this approach see Deco et al.^78^

The susceptibility measure of the whole-brain model was defined as the brain’s sensitivity to react to external stimulations as it was defined in previous works^38,39^. We computed the sensitivity of the perturbations on the spatiotemporal dynamics extended the definition of previous work, which determines the susceptibility in a system of coupled oscillators based on the response of the Kuramoto order parameter^55^. The Hopf model was perturbed for each G by randomly changing the local bifurcation parameter, *a*_*n*_, in the range [−0.02: 0]. The sensitivity of the perturbations on the spatiotemporal dynamics was calculated by measuring the modulus of the local Kuramoto order parameter as:

16 $${{\chi} =\big\langle {\big\langle {\big(\big\langle {\widetilde{{R}}_{{\lambda_{s}}}}({\bar{x}},t)\big\rangle}_{t}-{\big\langle {{R}_{{\lambda_{s}}}}(\bar{x},t)\big\rangle}_{t}\big)\big\rangle }_{{trials}}\big\rangle }_{\bar{x}}$$

where ${\widetilde{R}}_{{{{\lambda }}}_{s}}\left(\bar{x},t\right)$ corresponds to the perturbed case, the ${R}_{{{{\lambda }}}_{s}}\left(\bar{x},t\right)$ to the unperturbed case, and ${\left\langle \right\rangle }_{t}$, ${\left\langle \right\rangle }_{{trials}}$ and ${\left\langle \right\rangle }_{x}$ to the average across time, trials, and space, respectively.

The information encoding capability measures the ability of the system to encode external inputs, and such is closer related to complexity measures such as Lempel-Ziv (LZ) (used in Massimini seminal works^10,12^) or automatic complexity evaluator (ACE), and synchrony coalition entropy (SCE) (used and defined in^95^). The information capability, I, was defined as the standard deviation across trials of the difference between the perturbed ${\widetilde{R}}_{{\lambda }_{s}}(\bar{x},t)$ and unperturbed ${R}_{{{{\lambda }}}_{s}}\left(\bar{x},t\right)$ mean of the modulus of the local Kuramoto order parameter across time *t*, averaged across all brain areas *n* as:

17 $$I=\sqrt{{\big\langle {\big\langle {{\big(\big\langle {\widetilde{{R}}_{{\lambda_{s}}}}\big(\bar{x},t\big)\big\rangle }_{t}-{\big\langle {{R}_{{\lambda_{s}}}}\big(\bar{x},t\big)\big\rangle }_{t}\big)}^{2}\big\rangle }_{{trials}}\big\rangle }_{\bar{x}}-{\big\langle {{\big\langle {\big(\big\langle {\widetilde{{R}}_{{\lambda_{s}}}}\big(\bar{x},t\big)\big\rangle }_{t}-{\big\langle {{R}_{{\lambda_{s}}}}\big(\bar{x},t\big)\big\rangle }_{t}\big)\big\rangle }^{2}}_{{trials}}\big\rangle }_{\bar{x}}}$$

where the brackets ${\left\langle \right\rangle }_{t}$, ${\left\langle \right\rangle }_{{trials}}$ and ${\left\langle \right\rangle }_{x}$ denote the averages defined as above.

### Statistical analyses

We applied the Wilcoxon rank-sum method to test the differences between conditions in Kuramoto amplitude turbulence, information capacity, transfer correlation, and perturbative measures. For the node-level analysis, we applied the Kolmogorov–Smirnov test to compare between conditions. Additionally, we applied the False Discovery Rate (FDR) at the 0.05 level of significance to correct multiple comparisons^96^.

### Reporting summary

Further information on research design is available in the Nature Research Reporting Summary linked to this article.

## Supplementary information

Supplementary Information

Description of Additional Supplementary Files

Video S1

Video S2

Video S3

Video S4

Video S5

Video S6

Video S7

Reporting summary

## Data Availability

Sleep and meditation time-series are publicly available on https://github.com/aescrichs/brainstates-turbulence/releases. The disorders of consciousness datasets contain information from a clinical population and are not publicly available due to constraints imposed by the currently approved ethics protocol, however the data can be requested to the Authors.

## Code availability

All code written in support of this is publicly available on https://github.com/aescrichs/brainstates-turbulence.
