# Supplementary material for: Unifying turbulent dynamics framework distinguishes different brain states
Source: Commun Biol. 2022 Jun 29;5:638. doi: 10.1038/s42003-022-03576-6 (PMC9243255; doi:10.1038/s42003-022-03576-6)
Supplement: Supplementary file 1 — Supplementary Information [file 42003_2022_3576_MOESM1_ESM.pdf]

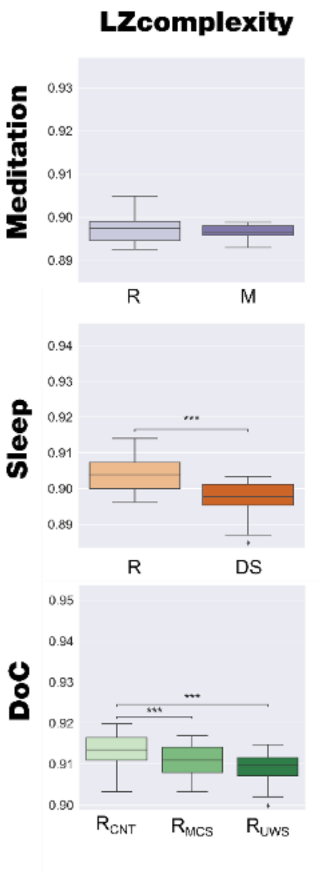

Supplementary Figure 1. Alternative complexity measure after perturbation. *We computed the algorithmic complexity through the Lempel-Ziv complexity of the system when it is perturbed for different brain states. To this end, we create a binary spatiotemporal distribution by z-scored the simulated times series after perturbation, ts(n,t), where if* $ts_{zscore}(n,t)>2=1$ *and if* $ts_{zscore}(n,t)<2=0$ *and computed the normalized complexity as* $\widetilde{C_L} = C_L \frac{\log_2(L)}{LH(L)}$ *where H(L) is the source entropy of a sequence of length L . We found that the complexity decreases for deep sleep, DOC patients similar to the results information encoding capability. Nevertheless, this metric is less sensitive than the one proposed in our work regarding disentangling between* R_{MCS} *and* R_{UWS} *and meditative state.*

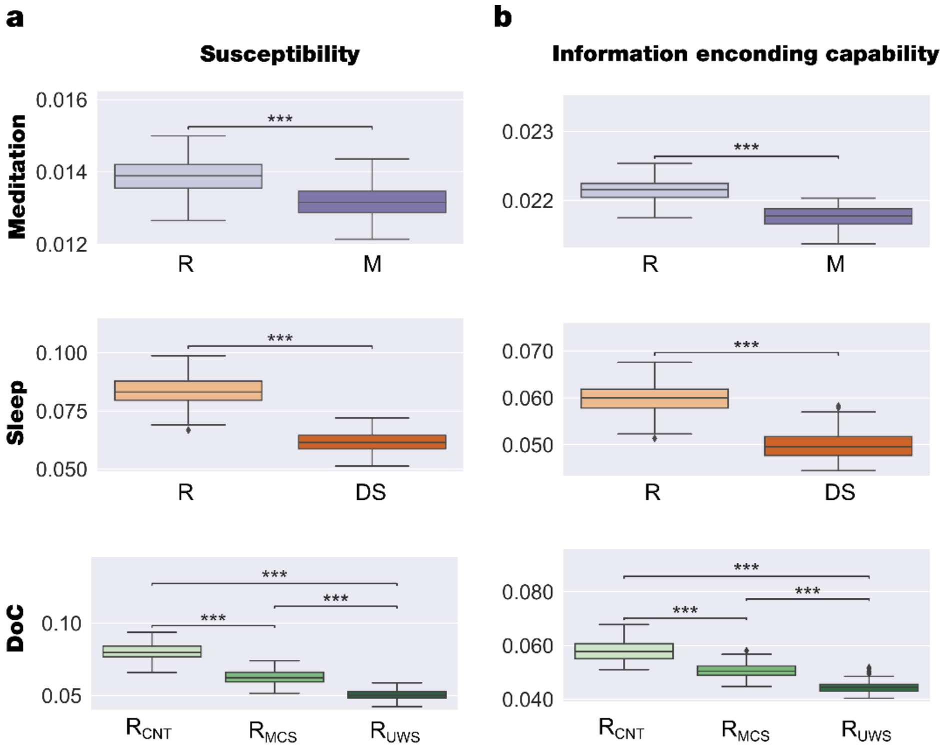

Supplementary Figure 2. Alternative implementation of perturbation reproduces main results. *We applied an external perturbation by randomly changing the bifurcation parameter of each brain area,* a_n*, within the range [-0.02:0]. We computed the susceptibility and information encoding capability after this alternative perturbation, and we found the resting state was the most sensitive in both metrics as we have found with the original perturbation.*
