# Supplementary material for: Unifying turbulent dynamics framework distinguishes different brain states
Source: Commun Biol. 2022 Jun 29;5:638. doi: 10.1038/s42003-022-03576-6 (PMC9243255; doi:10.1038/s42003-022-03576-6)
Supplement: Supplementary file 2 — Description of Additional Supplementary Files [file 42003_2022_3576_MOESM2_ESM.docx]

**Description of Additional Supplementary Files**

**File name**: Video S1

**Description:** Spatiotemporal evolution of turbulence in empirical data for the meditation dataset. The spatiotemporal evolution of turbulence in the empirical data from a single participant during resting state is summarised in the movie. The video visualises the change over time and space of the local Kuramoto order parameter, R, reflecting turbulence in the brain. The video shows the inflated 3D side and midline views as well as flat maps of the hemispheres over the time points.

**File name:** Video S2

**Description:** Spatiotemporal evolution of turbulence in empirical data for the meditation dataset. The spatiotemporal evolution of turbulence in the empirical data from a single participant during meditation is summarised in the movie. The video visualises the change over time and space of the local Kuramoto order parameter, R, reflecting turbulence in the brain. The video shows the inflated 3D side and midline views as well as flat maps of the hemispheres over the time points.

**File name**: Video S3

**Description**: Spatiotemporal evolution of turbulence in empirical data for the sleep dataset. The spatiotemporal evolution of turbulence in the empirical data from a single participant during wakefulness is summarised in the movie. The video visualises the change over time and space of the local Kuramoto order parameter, R, reflecting turbulence in the brain. The video shows the inflated 3D side and midline views as well as flat maps of the hemispheres over the time points

**File name**: Video S4

**Description**: Spatiotemporal evolution of turbulence in empirical data for the sleep dataset. The spatiotemporal evolution of turbulence in the empirical data from a single participant during deep sleep is summarised in the movie. The video visualises the change over time and space of the local Kuramoto order parameter, R, reflecting turbulence in the brain. The video shows the inflated 3D side and midline views as well as flat maps of the hemispheres over the time points

**File name**: Video S5

**Description**: Spatiotemporal evolution of turbulence in empirical data for the DOC dataset. The spatiotemporal evolution of turbulence in the empirical data from a healthy single participant is summarised in the movie. The video visualises the change over time and space of the local Kuramoto order parameter, R, reflecting turbulence in the brain. The video shows the inflated 3D side and midline views as well as flat maps of the hemispheres over the time points

**File name**: Video S6

**Description**: Spatiotemporal evolution of turbulence in empirical data for the DOC dataset. The spatiotemporal evolution of turbulence in the empirical data from a RMCS single participant is summarised in the movie. The video visualises the change over time and space of the local Kuramoto order parameter, R, reflecting turbulence in the brain. The video shows the inflated 3D side and midline views as well as flat maps of the hemispheres over the time points.

**File name**: Video S7

**Description**: Spatiotemporal evolution of turbulence in empirical data for the DOC dataset. The spatiotemporal evolution of turbulence in the empirical data from a RUWS single participant is summarised in the movie. The video visualises the change over time and space of the local Kuramoto order parameter, R, reflecting turbulence in the brain. The video shows the inflated 3D side and midline views as well as flat maps of the hemispheres over the time points.
