# Supplementary material for: Unifying turbulent dynamics framework distinguishes different brain states
Source: Commun Biol. 2022 Jun 29;5:638. doi: 10.1038/s42003-022-03576-6 (PMC9243255; doi:10.1038/s42003-022-03576-6)
Supplement: Supplementary file 10 — Reporting summary [file 42003_2022_3576_MOESM10_ESM.pdf]

nature portfolio

Corresponding author(s): AE; YS; MLK; GD

Last updated by author(s): Nov 18, 2021

Reporting Summary

Nature Portfolio wishes to improve the reproducibility of the work that we publish. This form provides structure for consistency and transparency in reporting. For further information on Nature Portfolio policies, see our Editorial Policies and the Editorial Policy Checklist.

Statistics

For all statistical analyses, confirm that the following items are present in the figure legend, table legend, main text, or Methods section.

n/a	Confirmed	
☐	☒	The exact sample size (*n*) for each experimental group/condition, given as a discrete number and unit of measurement
☐	☒	A statement on whether measurements were taken from distinct samples or whether the same sample was measured repeatedly
☐	☒	The statistical test(s) used AND whether they are one- or two-sided<br>*Only common tests should be described solely by name; describe more complex techniques in the Methods section.*
☐	☒	A description of all covariates tested
☐	☒	A description of any assumptions or corrections, such as tests of normality and adjustment for multiple comparisons
☐	☒	A full description of the statistical parameters including central tendency (e.g. means) or other basic estimates (e.g. regression coefficient) AND variation (e.g. standard deviation) or associated estimates of uncertainty (e.g. confidence intervals)
☐	☒	For null hypothesis testing, the test statistic (e.g. *F*, *t*, *r*) with confidence intervals, effect sizes, degrees of freedom and *P* value noted<br>*Give P values as exact values whenever suitable.*
☒	☐	For Bayesian analysis, information on the choice of priors and Markov chain Monte Carlo settings
☒	☐	For hierarchical and complex designs, identification of the appropriate level for tests and full reporting of outcomes
☒	☐	Estimates of effect sizes (e.g. Cohen's *d*, Pearson's *r*), indicating how they were calculated

Our web collection on statistics for biologists contains articles on many of the points above.

Software and code

Policy information about availability of computer code

Data collection	All data were acquired using proprietary software from MRI
Data analysis	Matlab R2021a

For manuscripts utilizing custom algorithms or software that are central to the research but not yet described in published literature, software must be made available to editors and reviewers. We strongly encourage code deposition in a community repository (e.g. GitHub). See the Nature Portfolio guidelines for submitting code & software for further information.

Data

Policy information about availability of data

All manuscripts must include a data availability statement. This statement should provide the following information, where applicable:

- Accession codes, unique identifiers, or web links for publicly available datasets
- A description of any restrictions on data availability
- For clinical datasets or third party data, please ensure that the statement adheres to our policy

Sleep and meditation time-series are publicly available on https://github.com/aescrichs/brainstates-turbulence. The disorders of consciousness datasets contain information from a clinical population and are not publicly available due to constraints imposed by the currently approved ethics protocol.

Field-specific reporting

Please select the one below that is the best fit for your research. If you are not sure, read the appropriate sections before making your selection.

☒ Life sciences ☐ Behavioural & social sciences ☐ Ecological, evolutionary & environmental sciences

For a reference copy of the document with all sections, see nature.com/documents/nr-reporting-summary-flat.pdf

Life sciences study design

All studies must disclose on these points even when the disclosure is negative.

Sample size	Sample sizes were determined as a compromise between adequate statistical power and limitations imposed during ethics review. The datasets included in this study have been previously analyzed from the perspective of resting state functional connectivity, with results strongly suggesting sufficient statistical power.
Data exclusions	Sleep<br>We selected 13 subjects who reached the deep sleep stage (DS, i.e., N3) and contiguous time series of at least 198 volumes<br><br>Disorders of consciousness: Paris<br>We excluded subjects with T1 acquisition errors (n=5), with high levels of motion detected (n=7), registration errors (n=4), and large focal brain lesions (n=4).<br><br>Disorders of consciousness: Liege<br>The exclusion criteria of patients were as follows: (i) having any significant neurological, neurosurgical or psychiatric disorders prior to the brain insult that lead to DOC, (ii) having any contraindication to MRI such as electronic implanted devices, external ventricular drain, and (iii) being not medically stable or large focal brain damage, i.e. >2/3 of one hemisphere.
Replication	Model-based results were replicated by 100 iteratios for each brain state condition.
Randomization	Randomization measures were not necessary for this data. For the sleep dataset, each subject contributed to the wake and deep sleep conditions, allowing for paired comparisons without the need for a randomized control group. Similarly, for the meditation dataset, each subject was scanned during resting-state and meditation. For the dataset of patients with disorders of consciousness, a control group was formed by selecting a group of age-matched controls.
Blinding	Investigators were not blinded during data collection and analyses.

Reporting for specific materials, systems and methods

We require information from authors about some types of materials, experimental systems and methods used in many studies. Here, indicate whether each material, system or method listed is relevant to your study. If you are not sure if a list item applies to your research, read the appropriate section before selecting a response.

Materials & experimental systems

n/a	Involved in the study	
☒	☐	Antibodies
☒	☐	Eukaryotic cell lines
☒	☐	Palaeontology and archaeology
☒	☐	Animals and other organisms
☐	☒	Human research participants
☒	☐	Clinical data
☒	☐	Dual use research of concern

Methods

n/a	Involved in the study	
☒	☐	ChIP-seq
☒	☐	Flow cytometry
☐	☒	MRI-based neuroimaging

Human research participants

Policy information about studies involving human research participants

Population characteristics	Meditators were recruited from Vipassana communities of Barcelona, Catalonia, Spain (7 females, mean ± SD, 39.8±10.29 years, 9,526.9±8,619.8 meditation experience).<br><br>Sleep dataset: 8 females, mean ± SD age of 23.1±2.6 years.<br><br>Disorders of consciousness, Paris: We included 33 patients in MCS (11 females, mean age ± SD, 47.25± 20.76 years), and 24 in UWS (10 females, mean age ± SD, 39.25± 16.30 years) and 13 healthy controls (7 females, mean age ± SD, 42.54± 13.64 years).

	Disorders of Consciousness Liege: We included 33 patients in MCS (9 females, mean age±SD, 45±16 years), and 15 in UWS (6 females, mean age±SD, 47±16 years).
Recruitment	Meditation: Meditators with more than 1000 hours of meditation experience were selected from Vipassana communities of Barcelona, Catalonia, Spain<br><br>Sleep dataset: Subjects were recruited by online advertisement.<br><br>Disorders of consciousness Liege. Patients were screened and recruited from the University Hospital in Liege, Belgium.<br><br>Disorders of consciousness: Paris. Patients were screened and recruited in Paris Pitié-Salpêtrière Hopital, France.
Ethics oversight	The protocols have been approved by the Ethics Committee of the Bellvitge University Hospital, the Ethics Committee of the Goethe-Universität Frankfurt, the Ethics Committee of the Medical School of the University of Liège, and the Ethics Committee of the Pitié-Salpêtrière Hospital. Informed consent to participate in the study was obtained from the healthy subjects and from the legal surrogates of the patients.

Note that full information on the approval of the study protocol must also be provided in the manuscript.

Magnetic resonance imaging

Experimental design

Design type	resting-state fMRI
Design specifications	*Specify the number of blocks, trials or experimental units per session and/or subject, and specify the length of each trial or block (if trials are blocked) and interval between trials.*
Behavioral performance measures	Clinical assessment and trained clinicians carried out the clinical evaluation and Coma Recovery Scale-Revised (CRS-R) scoring to determine the patients' state of consciousness.

Acquisition

Imaging type(s)	functional and structural MRI
Field strength	3T
Sequence & imaging parameters	Meditation<br>MRI images were acquired on a 3T Siemens Trio scanner (Siemens, Erlangen, Germany) using a 32-channel receiver coil. The high-resolution T1-weighted images were acquired with 208 contiguous sagittal slices; TR/TE= 1970 ms/ 2.34 ms; inversion time (IT) = 1050 ms; flip angle = 9°; FOV = 256 mm; and isotropic voxel size 1 mm. Resting-state and meditation fMRI images were performed by a single shot gradient-echo EPI sequence with a total of 450 volumes (15 min); TR/TE = 2000 ms/29 ms; FOV= 240 mm; in-plane resolution 3 mm; 32 transversal slices with thickness = 4 mm; flip angle =80°.<br><br>Sleep<br>MRI images were acquired on a 3-T Siemens Trio scanner (Erlangen, Germany). EEG via a cap (modified BrainCapMR, Easycap, Herrsching, Germany) was recorded continuously during fMRI acquisition (1505 volumes of T2-weighted echo planar images, TR/TE = 2080 ms/30 ms, matrix 64×64, voxel size 3×3×2 mm3, distance factor 50%; FOV 192 mm2).<br><br>Disorders of consciousness: Paris<br>MRI images were acquired with two different acquisition protocols. In the first protocol, MRI data were acquired on a 3T General Electric Signa System. T2*-weighted whole brain resting state images were acquired with a gradient-echo EPI sequence using axial orientation (200 volumes, 48 slices, slice thickness: 3 mm, TR/TE: 2400 ms/30 ms, voxel size: 3.4375× 3.4375 × 3.4375 mm, flip angle: 90°, FOV: 220 mm2 448).<br>In the second protocol, MRI data were acquired on a 3T Siemens Skyra System. T2*-weighted whole brain resting state images were recorded with a gradient-echo EPI sequence using axial orientation (180 volumes, 62 slices, slice thickness: 2.5 mm, TR/TE: 2000 ms/30 ms, voxel size: 2 × 2× 2 mm, flip angle: 90°, FOV: 240 mm2 454 , multiband factor: 2).<br><br>Disorders of consciousness: Liège<br>MRI images were acquired on a Siemens 3T Trio scanner (Siemens Inc, Munich, Germany). MRI acquisition included a gradient echo-planar imaging (EPI) sequence (32 transversal slices, 300 volumes, TR/TE = 2000 ms/30 ms, flip angle = 78°, voxel size = 3x3x3 mm, FOV = 192 mm); a structural T1 (120 transversal slices, TR = 2300 ms, voxel size = 1.0x1.0x1.2 mm, flip angle = 9°, FOV= 256 mm).
Area of acquisition	whole-brain

Diffusion MRI ☒ Used ☐ Not used

Parameters	We used the Human Connectome Project (HCP) database that contains diffusion spectrum and T2-weighted neuroimaging data from 32 participants as reported in Deco and Kringelbach (2020). A complete description of the acquisition parameters is described in detail on the HCP website (Setsompop, K. et al. 2013). The freely Lead DBS software package (https://www.lead-dbs.org/) provides the pre-processing described in detail in Horn et al. 2017. The data were processed by using a q-sampling imaging algorithm implemented in DSI studio (http://dsi-studio. labsolver.org). A white-matter mask was computed by segmenting the T2-weighted images and co-registering the images to the b0 image of the diffusion data using SPM12. For each HCP participant, 200,000 fibres were sampled within the white-matter mask. Fibres were transformed into MNI space using Lead-DBS Horn and Blankenburg (2016). Finally, we used the standardized methods in Lead-DBS to extract the structural connectomes from the Schaefer 100 parcellation.

Preprocessing

Preprocessing software	The pre-processing of resting-state data was computed using MELODIC (Multivariate Exploratory Linear Optimized Decomposition into Independent Components) which is part of FSL (http://fsl.fmrib.ox.ac.uk/fsl).
Normalization	Processing included normalization by using T1 image unified segmentation
Normalization template	Montreal Neurological Institute standard space (MNI)
Noise and artifact removal	Artifact and noise components were regressed out independently for each subject using FIX (FMRIB's ICA-based X-noiseifier)
Volume censoring	We discarded the first five fMRI volumes in each subject to allow for signal stabilization

Statistical modeling & inference

Model type and settings	Pearson's linear correlation between BOLD time series.
Effect(s) tested	n/a (no task was performed in the experiments)

Specify type of analysis: ☐ Whole brain ☐ ROI-based ☒ Both

Anatomical location(s)	1000 ROIs were defined based on the Schaefer parcellation atlas, covering the whole-brain network.
Statistic type for inference (See Eklund et al. 2016)	We did not perform voxel-wise inference
Correction	False Discovery rate correction

Models & analysis

n/a	Involved in the study
☐	☒ Functional and/or effective connectivity
☒	☐ Graph analysis
☒	☐ Multivariate modeling or predictive analysis

Functional and/or effective connectivity	Functional connectivity was computed as the pairwise Pearson's correlation coefficient between regional BOLD signals.
